# Editorial for the Special Issue on AC Electrokinetics in Microfluidic Devices

**DOI:** 10.3390/mi10050345

**Published:** 2019-05-25

**Authors:** Antonio Ramos, Pablo García-Sánchez

**Affiliations:** Departamento de Electrónica y Electromagnetismo, Facultad de Física, Universidad de Sevilla, Avda. Reina Mercedes s/n, 41012 Sevilla, Spain

The use of AC electric fields for manipulating and/or characterizing liquids and small particles in suspension is well-known. Owing to miniaturization, several applications in microsystems have appeared over the last few decades in multiple research fields, such as colloidal science, microelectronics and biotechnology. For example, dielectrophoretic (DEP) forces can be used for manipulation and separation of a great variety of particles, such as biological cells, semiconductor nanowires or tiny metal colloids. DEP combined with electrokinetic-induced fluid flows can be leveraged for particle concentration in microfluidic devices. Additionally, application of AC fields gives rise to particle–particle interactions that lead to self-assembly patterns, a common bottom-up approach for the fabrication of engineered microstructures. A number of electric field-induced fluid flows occur in microelectrode structures; these flows can be used for standard liquid manipulation such as pumping and mixing. In addition, the electrical control of the substrate wettability can be achieved by the electrowetting effect, allowing for fine tuning of contact angle and droplet manipulation within microsystems. Besides particle manipulation, AC electrokinetics has also been used to characterize the dielectric properties of particles through DEP, as well as to assist other measurement techniques such as fluorescent spectroscopy or electrical impedance spectroscopy by pre-concentrating the particles.

This Special Issue contains nine research paper and one review article on the following aspects of electrokinetics and microfluidics: (1) Applications of AC electrokinetic flows, (2) Electric field manipulation of cells, (3) Finite-ion-size effects on Electrokinetics, (4) Electrokinetics of semiconductors, (5) Electrowetting, (6) Electroconvective instabilities and (7) Liquid crystal-enabled electrokinetics.

1. Hu et al. [1] reported a numerical study of asymmetrical ICEO flows on a herringbone floating electrode and developed a novel micromixer. Miloh [2] theoretically analyzed the possibility of using ICEO and DEP for controlling the location of quantum dots near an ellipsoidal nano-antenna. Also, numerical simulations by Salari and Dalton [3] demonstrated simultaneous mixing and pumping by means of a double-array AC electrothermal device consisting of two opposing microelectrode arrays.
2. Lo and Lei [4] showed a simple flow-through device for continuous and massive lysis of cells via electricity. Banovetz et al. [5] reported a method that uses an array of wireless bipolar electrodes to pattern cells into clusters.
3. The influence of finite ionic size on the dielectric and electrokinetic properties of colloidal suspensions is analyzed by López-García et al. [6]. They extended previous works to systems made of three or more ionic species with different ionic sizes and found qualitative and quantitative differences with respect to the standard electrokinetic model.
4. García-Sánchez et al. [7] theoretically studied the ac electrokinetic response of semiconducting microspheres immersed in an electrolyte.
5. Chamakos et al. [8] combined experiments and simulations to study the effect of the solid topography and dielectric thickness on the dynamics of electrostatically-induced spreading of a liquid drop on a solid surface.
6. Kim et al. [9] used numerical simulations to show that electroconvection can appear near flat inert electrodes for oscillatory applied electric fields.
7. Electrokinetic phenomena are usually studied within isotropic electrolytes. A new mechanism for spatial charge generation occurs when this isotropic medium is replaced by a liquid crystal. The review by Peng and Lavrentovich [10] presents the main features of liquid crystal-enabled electrokinetics.

We sincerely thank all authors for submitting their manuscripts to this Special Issue. We would also like to acknowledge all the reviewers for their dedication to provide careful and timely reviews that ensure the quality of this Special Issue.

## References

[1] Hu Q., Guo J., Cao Z., Jiang H. (2018). Asymmetrical Induced Charge Electroosmotic Flow on a Herringbone Floating Electrode and Its Application in a Micromixer. Micromachines.

[2] Miloh T. (2019). AC Electrokinetics of Polarizable Tri-Axial Ellipsoidal Nano-Antennas and Quantum Dot Manipulation. Micromachines.

[3] Salari A., Dalton C. (2019). Simultaneous Pumping and Mixing of Biological Fluids in a Double-Array Electrothermal Microfluidic Device. Micromachines.

[4] Lo Y.J., Lei U. (2019). A Continuous Flow-through Microfluidic Device for Electrical Lysis of Cells. Micromachines.

[5] Banovetz J.T., Li M., Pagariya D., Kim S., Ganapathysubramanian B., Anand R.K. (2019). Defining Cell Cluster Size by Dielectrophoretic Capture at an Array of Wireless Electrodes of Several Distinct Lengths. Micromachines.

[6] López-García J., Horno J., Grosse C. (2018). Numerical Solution of the Electrokinetic Equations for Multi-ionic Electrolytes Including Different Ionic Size Related Effects. Micromachines.

[7] García-Sánchez P., Flores-Mena J.E., Ramos A. (2019). Modeling the AC Electrokinetic Behavior of Semiconducting Spheres. Micromachines.

[8] Chamakos N.T., Sema D.G., Papathanasiou A.G. (2019). Highlighting the Role of Dielectric Thickness and Surface Topography on Electrospreading Dynamics. Micromachines.

[9] Kim J., Davidson S., Mani A. (2019). Characterization of Chaotic Electroconvection near Flat Inert Electrodes under Oscillatory Voltages. Micromachines.

[10] Peng C., Lavrentovich O.D. (2019). Liquid Crystals-Enabled AC Electrokinetics. Micromachines.

